# High level of H3K4 tri-methylation modification predicts poor prognosis in esophageal cancer

**DOI:** 10.7150/jca.36801

**Published:** 2020-03-05

**Authors:** Xu-Dong Ye, Bai-Quan Qiu, Dian Xiong, Xu Pei, Na Jie, Hua Xu, Shu-Qiang Zhu, Xiang Long, Zheng Xu, Hai-Bo Wu, Jian-Jun Xu, You-Sheng Huang, Yong-Bing Wu

**Affiliations:** 1 Department of Cardiothoracic Surgery, The Second Affiliated Hospital of Nanchang University, Jiangxi Province 330000, P. R. China.; 2 Department of Thoracic Surgery, The Central Hospital of Xuhui District, Shanghai, 20031, P. R. China; 3 Department of Pathology, The First Affiliated Hospital of Hainan Medical University, Hainan Medical University, Haikou, Hainan 571101, P.R. China

**Keywords:** H3K4me3, HEC, prognosis

## Abstract

**Objectives**: An increase in the trimethylation of lysine 4 of histone 3 (H3K4me3) has been reported to be involved in the development of several types of tumors. However, the level and role of H3K4me3 in human esophageal cancer (HEC) remain unknown. Here, we assessed the role and clinical significance of H3K4me3 in HEC.

**Methods**: The level of H3K4me3 was determined in 15 pairs of HEC and paracancerous tissues by Western blotting. A tissue microarray including samples from 100 HEC patients was analyzed by immunohistochemistry to determine the relationship between the level of H3K4me3 and the clinicopathological features of HEC patients. Then, the levels of H3K4me3 in HEC cells were elevated via knockdown of inhibitor of growth family member 4(Ing4) expression. Finally, the prognostic significance of H3K4me3 levels in HEC patients was further analyzed.

**Results**: We found that H3K4me3 levels were frequently elevated in HEC tissues compared with adjacent esophageal tissues, and elevated H3K4me3 was significantly associated with poor tumor differentiation (p =1.39×10-5) and advanced tumor stage (p=8.5×10-5). After Ing4 knockdown in HEC cells, we found that the cell proliferation, metastasis, invasion and colony formation abilities were enhanced compared to those in the control cells. Notably, we found that HEC patients with a high level of H3K4me3 exhibited an unfavorable 5-year survival rate compared to those with a low level of H3K4me3 (p=6.8×10-5). The univariate analysis showed that the tumor differentiation, TNM stage, and H3K4me3 level were predictors of the overall survival rate of HEC patients. In the multivariate analysis, tumor stage (p=0.015) and H3K4me3 level (p=0.034) were revealed to be independent parameters for predicting the prognosis of HEC patients.

**Conclusions**: Thus, high levels of H3K4me3 may be used as a meaningful biomarker for HEC prognosis evaluation.

## Introduction

Human esophageal cancer (HEC) is the sixth leading cause of cancer-related death worldwide 1. Currently, the most important and most effective treatment option for HEC patients relies strongly on early diagnosis 2. Although the management of patients with esophageal cancer has greatly improved over the past few decades, the overall survival (OS) rate of HEC patients is still low, the 5-year survival rate of HEC patients is less than 10%, and the 5-year survival rate of esophageal cancer patients who undergo postoperative resection is less than 40% 3. Additionally, HEC patients who undergo surgical treatment still have high recurrence rates 4. Thus, it is urgent to reveal the mechanism of HEC progression.

The occurrence of cancer is a multistep, accumulative process, and cancer is a genetic disease. It is widely accepted that not only somatic mutations but also epigenetic changes contribute to this process; epigenetic changes alter chromatin structure leading to downregulation of gene expression 5. In fact, epigenetic dysregulation is closely related to tumorigenesis 6, 7, and has recently attracted increasing attention because it is intimately related to metabolic reprogramming, one of the emerging hallmarks of cancer 8. Epigenetic modifications in cancer mainly include DNA methylation and histone modification. Indeed, histone modification has been identified to regulate a wide range of critical biological processes, such as cell proliferation, apoptosis, and cell cycle 9. Multiple studies in recent years have demonstrated that changes in histone epigenome are one of the early procedures in oncogene formation 10. For example, the dysregulation of genome-wide mapping of chromatin had been revealed during cancer initiation and progression due to advances in high-throughput sequencing. The abnormal trimethylation of lysine 4 on histone H3 (H3K4me3) has been found in breast cancer 11, colon cancer 12, pancreatic cancer 13 and is associated with poor prognosis of malignant tumors 14, 15. Additionally, ING4, the reader of the chromatin H3K4me3, has been identified to a be tumor suppressor gene 16, 17. Thus, the dysregulation of histone modification plays an important role in cancer.

This study aimed to identify the prognosis value of hypermethylation on histone H3K4 in patients with HEC. First, we assessed the H3K4me3 modification in HEC and matched paratumor tissues. By Ing4 knockdown, the roles of H3K4me3 in HEC was indirectly determined. Finally, the clinical significance of H3K4me3 in HEC was analyzed.

## Materials and Methods

### Patients and specimens

Fifteen HEC tissues were randomly collected from 100 patients by curative resection at the Affiliated Hospital of Hainan Medical University (Haikou, China) and the Second Affiliated Hospital of Nanchang University (Nanchang, China) between January 2005 and January 2006. Samples' collection and preservation is consistent with our previous study 18. Before surgery, no patients received any form of radiotherapy or chemotherapy, and detailed clinicopathological data were obtained from the patients. The clinical stage of the patients was evaluated by the TNM staging system of the American Joint Committee on Cancer (AJCC) and IUCC (8th edition) 19. All patients were routinely followed-up with clinical examination and thoracoabdominal CT every six months and with upper gastrointestinal endoscopy at two years. The median follow-up period was 43 months (range, 1-66 months) and the last follow-up was in July 2010. The overall survival (OS) was defined as the time interval between surgery to death or the last visit to the patient. The approval of ethics was approved by the Ethics Committee of the Second Affiliated Hospital of Nanchang University and the First Affiliated Hospital of Hainan Medical University.

### Western blot analysis

Total protein was prepared with RIPA buffer (Beyotime, Shanghai, China) and protein concentration was determined using a BCA kit (Beyotime, Shanghai, China). Thirty µg of protein were used in western blot analysis according to our previous study 18. The rabbit anti-human monoclonal H3K4 antibody (#9751, 1:1000 dilution, Cell Signaling Technology) and a mouse anti‑human monoclonal histone 3 antibody (1:1000 dilution, Beyotime, China) were employed in our study. Detection was routinely performed with a chemiluminescent HRP substrate (Beyotime, Shanghai, China) and an electrogenerated chemiluminescence (ECL) imaging system (Tanon, Shanghai, China).

### Tumor microarray construction and Immunohistochemistry

The construction process and detailed information on the HEC tissue microarray (TMA) and immunohistochemistry (IHC) process have been described previously 24,25.Briefly, the slides were deparaffinized and rehydrated in xylene and alcohol gradient, then treated with citric acid epitope retrieval reagent at 100°C for 20 min and cooled to room temperature to prohibit the endogenous peroxidase activity. To block nonspecific binding sites, the sections were incubated with 5% bovine serum albumin (BSA) (YESEN, Shanghai, China) at 37˚C for 30 min. Subsequently, the sections were incubated with primary rabbit anti‑human monoclonal H3K4me3 antibody (#9751, 1:200 dilution, Cell Signaling Technology) overnight at 4°C. The sections were washed with PBS three times and then incubated for 1 hours with horseradish peroxidase (HRP)-labeled secondary antibody (Gene Tech; Shanghai, China). Finally, the sections were stained using diaminobenzidine (DAB) (Gene Tech; Shanghai, China) and imaged by using a microscope (Leica Microsystems Imaging Solutions, Cambridge, UK).

### Evaluation of immunostaining intensity of TMAs

The positive expression of H3K4me3, which was immunohistochemically stained yellow or brown in the nucleus, was based on the combination of both intensity scores (ranging from 0 to 3 representing no, weak, moderate, and strong staining, respectively) and the percentage of positive tumor cells (0,1≤25%; 2, 25%-50%; 3, 50%-75%; 4, >75%). The combined scores ranged from 0 to a maximum of 7. A cutoff of ≤3 was considered the low H3K4me3level, and higher scores were considered high H3K4me3level.

### Lentivirus construction and transfection

Genomeditech (Shanghai, China) designed and supplied Ing4-NC and Ing4-shRNA (designs the target shRNA sequence) lentiviruses to us (**Table S1).** The lentiviral vectors were transfected HEC cells according to the manufacture instructions.

### Cell proliferation, colony formation, wound healing and transwell assay

Cell proliferation, colony formation, wound healing and transwell assays were performed according to the manufacturer's protocol and our past reports^20^, ^21^.

### Statistical analysis

Analyses were performed using SPSS 21.0 software (SPSS, Chicago, IL) and PRISM 5.0 (GraphPad Software Inc., San Diego, CA, USA). Values are expressed as the mean ± standard deviation. The Student *t* test was used for the comparison of H3K4me3 level in HEC and para-cancerous specimens. The relationship between categorical variables and the H3K4me3 level were analyzed by Chi-square (*χ*2) test. The overall survival (OS) rate of HEC patients was determined by Kaplan-Meier method and log-rank test. A multivariate analysis of the Cox proportional hazard regression model was used to analyze the independent prognostic factors. Beta (regression coefficient) represented the coefficient of low hazards factor compared with the high one. *P* < 0.05 was regarded as the statistical significance, and * indicates *p*< 0.05, ** indicates *p*< 0.01, and *** indicates *p*< 0.001.

## Results

### The level of H3K4me3 is elevated in human esophageal cancer tissues

**Figure 1:**
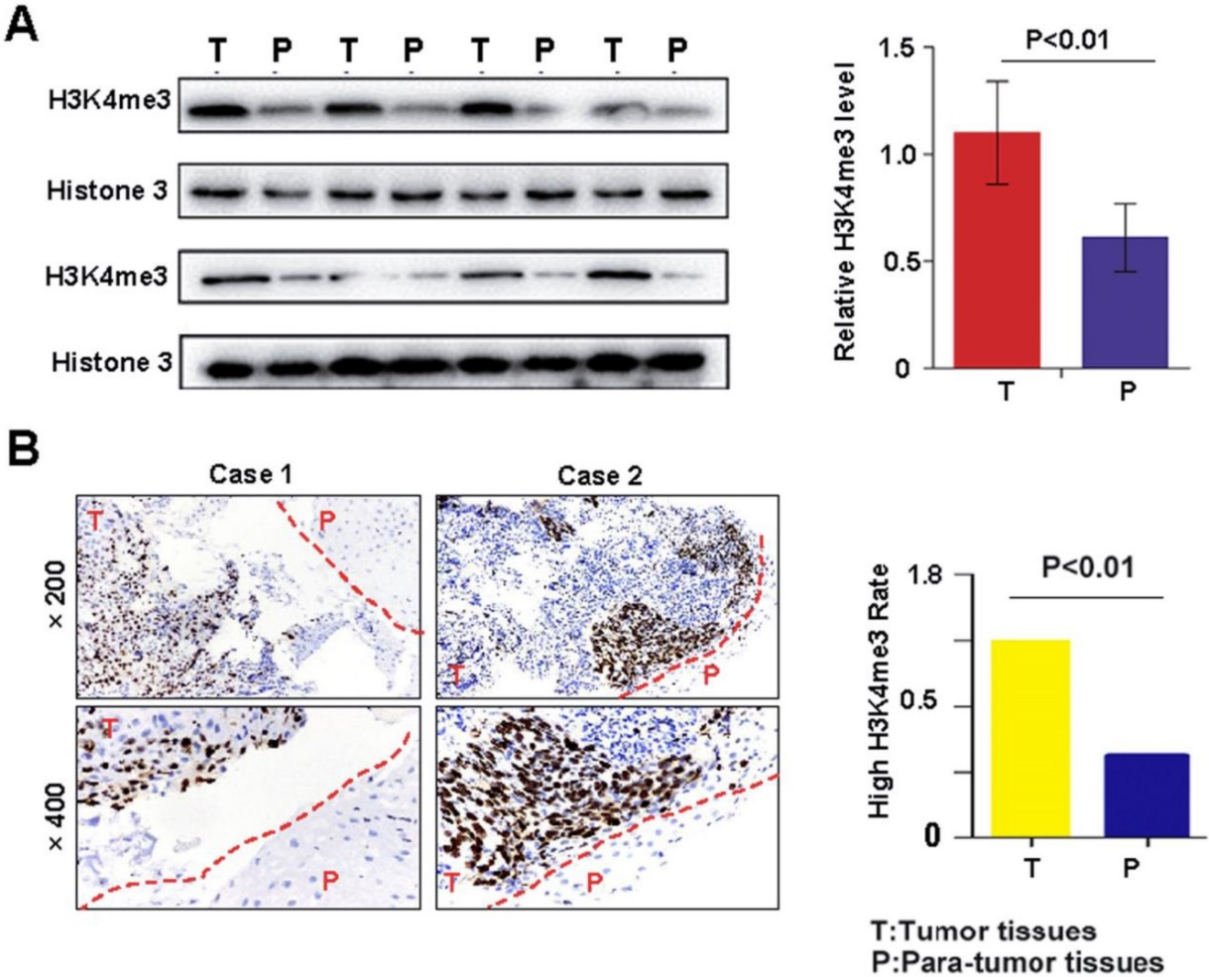
** H3K4me3 levels in HEC tissues and their corresponding adjacent normal esophagus tissues. A.** The protein expression of H3K4me3 in 15 paired HEC tissues (T) and adjacent non-tumor tissues (P) by Western blotting*P < 0.05**. B.** Representative column chart of H3K4me3 protein expression in 15 paired HEC tissues matched adjacent normal tissue. **C.** By immunohistochemistry, H3K4me3 staining was localized to the nucleus, and the level of H3K4me3 in HEC tissues was evidently stronger than that in their corresponding adjacent normal esophagus tissues. **D.** In tissue microarray, the proportion of HEC tissues with high H3K4me3 levels, 65% (65/100), was higher than that in adjacent normal esophageal tissues.

Western blotting and immunohistochemistry were performed to detect the level of H3K4me3 in HEC tissues and matched adjacent non-tumor tissues. The H3K4me3 level was significantly increased in HEC compared with that in corresponding adjacent normal esophageal tissues (1.10±0.24 vs. 0.61±0.16; **Fig. 1A**). Immunohistochemical analysis demonstrated that the intensity H3K4me3 staining was markedly higher in HEC tissues than in paratumor tissues (**Fig. 1B**). Overall, the elevated level of H3K4me3 immunostaining was more frequent in tumor tissues (60%, 60/100) than in matched non-tumor tissues (40%, 40/100; **Fig. 1B**). Immunohistochemically, the H3K4me3 staining was mainly in the nuclear and, to a lesser degree, the cytoplasmic in HEC tissues. The aforementioned results indicate that H3K4me3 may contribute to the onset and progression of HEC.

### Association between H3K4me3 and clinicopathological features of HECs

**Figure 2:**
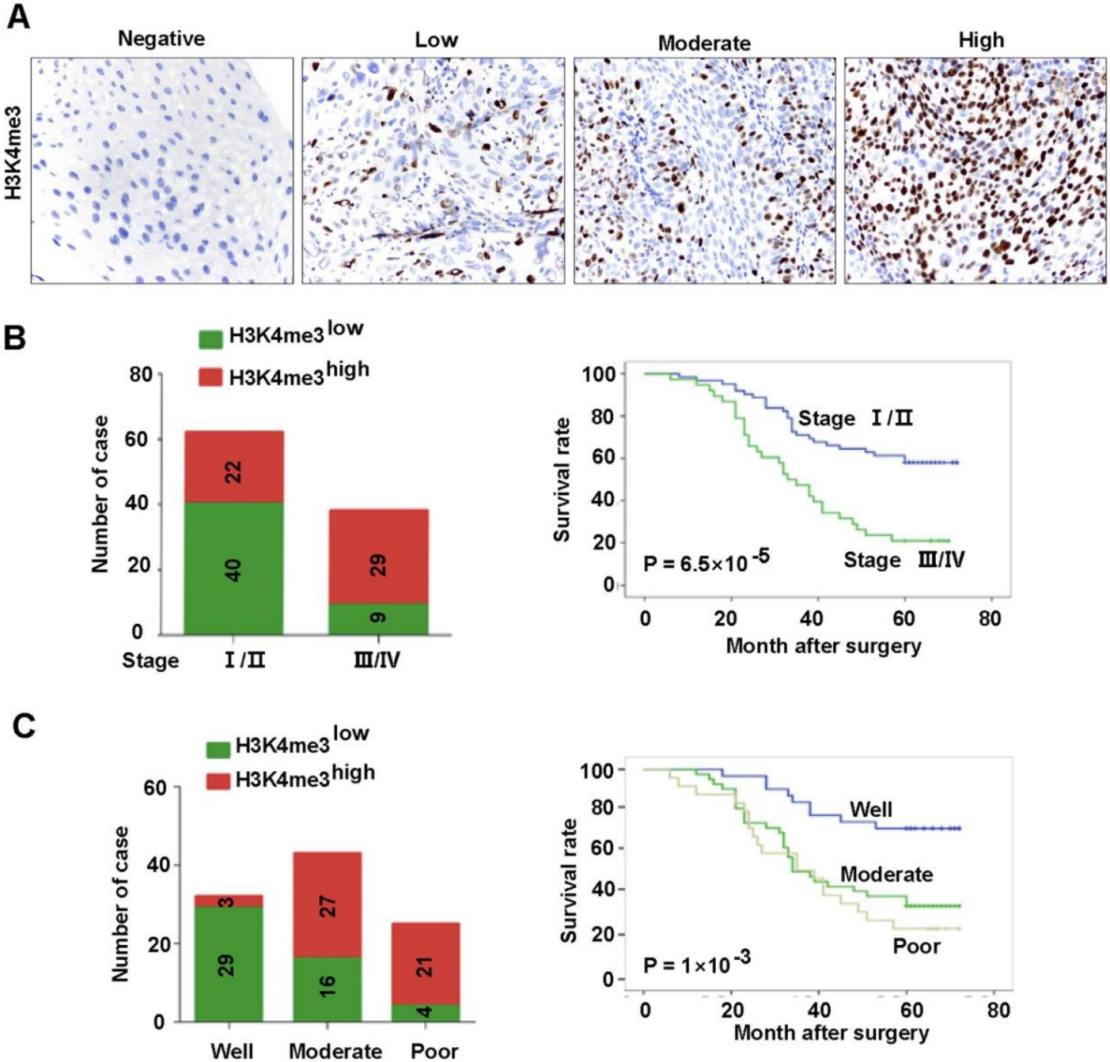
** Association between H3K4me3 level and the clinicopathological parameters of HEC patents. A.** Representative images showed the intensity of immunostaining. **B.** Patients with different TNM stages had different H3K4me3 levels, and evidently different overall survivals. **C.** Patients with different differentiation levels had different levels of H3K4me3, and had evidently different overall survivals.

The association between the clinic-pathological features of the patients and H3K4me3 was analyzed. Detailed clinical and pathological information was presented in our previous study 18. Briefly, a total of 100 cases of primary HEC was included in our analysis, of which 27 (27%) were women and 73 (73%) were men. There were 62 (62%) tumors in TNM stages I-II and 38 (38%) tumors in stages III-IV. In addition, the number of tumors with low and high differentiation was 25 and 75, respectively. The association between H3K4me3 level and clinicopathological parameters was listed in **Table 1** and **Fig. 2**. The H3K4me3 levels in HEC tissues varies greatly (**Figure 2A**), and we dichotomized them into H3K4me3 high (moderate and strong; n =51) and H3K4me3 low (negative and weak; n =49) groups. H3K4me3 levels were positively associated with high TNM stage (P=8.5×10^-5^), and poor tumor differentiation (P= 1.39×10^-5^). Tumors with high H3K4me3 levels were usually be detect in TNM stage III-IV (76.3%, 29/38), but not in TNM stage I-II (35.4%, 22/62; **Fig. 2B**). Furthermore, high H3K4me3 were positively associated with the tumor differentiation (84%, 21/25 vs. 40%, 30/75; **Fig. 2C**). However, other clinicopathological features, including age and gender, were not associated with the level of H3K4me3.

### Elevated H3K4me3 promote the HEC progression *in vitro*

**Figure 3:**
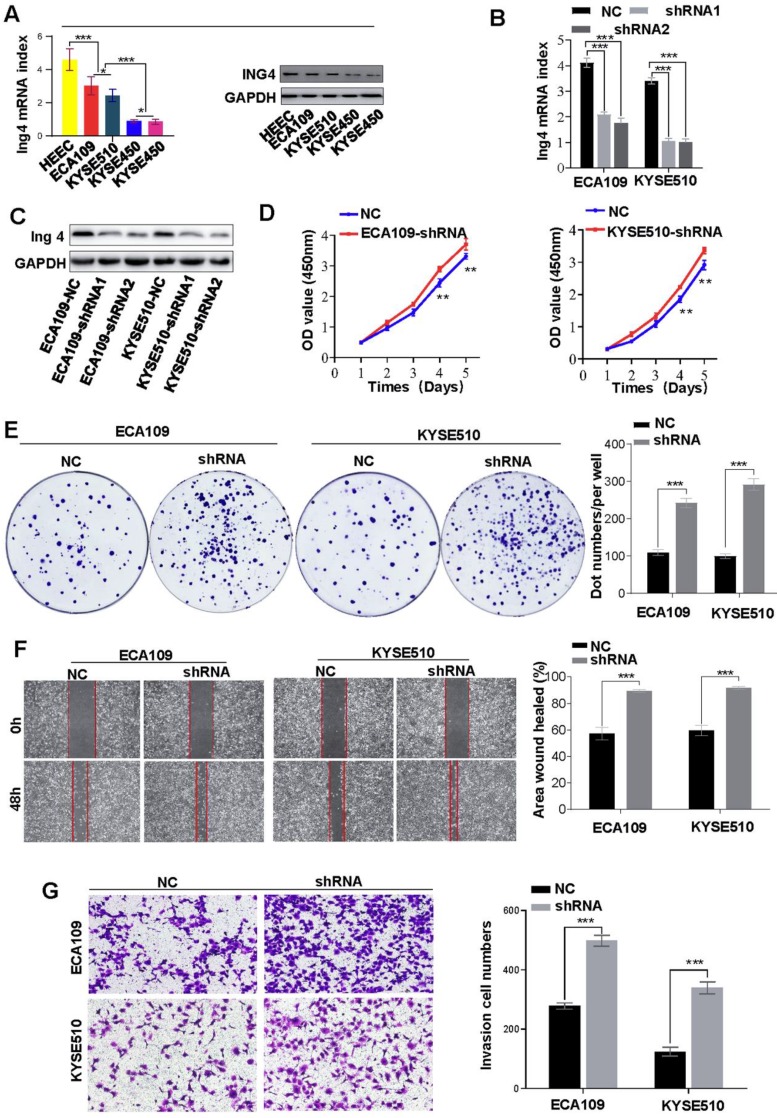
** Knockdown of Ing4 promotes HEC cell proliferation, metastasis and invasion. A.** The expression of Ing4 in HEC cells was determined by qRT-PCR and western blot. **B and C.** Ing4 was satisfactorily knock down in ECA109 and KYSE510 cells. **D.** The proliferation of ECA109- and KYSE510-shIng4 and the control cells was determined by CCK-8 assay (** < 0.05). **E.** Colony formation assay showed that the colonies formed by ECA109- and KYSE510-shIng4 cells were fewer than those formed by the control groups. **F.** A scratch test showed that the knockdown of Ing4 enhanced the cell migration. **G.** The invasion assay showed that the invasion of ECA109-shIng4 and KYSE510-shIng4 cell was markedly increased compared to their control cells.

Previously, Ing4 was reported to be a reader of H3K4me3 and broker crosstalk between H3K4 methylation and histone H3 acetylation. Here, we investigated the functions of H3K4me3 by interfering with Ing4 expression. After successfully knockdown of Ing4 expression (**Figures 3A-C**), we found that the proliferation of ECA109 and KYSE510 was enhanced (**Figure 3D**). In the colony formation assay, the colonies formed by ECA109-shIng4 and KYSE510-shIng4 cells were more abundant than those formed by ECA109- and KYSE510-NC groups (**Figure 3E**)**.** Moreover, metastasis and invasion were also promoted by Ing4 knockdown in HEC cells (**Figures 3F and 3G**). Thus, our results indirectly revealed that a high level of H3K4me3 promoted HEC progression.

### High level of H3K4me3 was associated with poor prognosis of patients with HEC

Patients with high H3K4me3 levels exhibited a worse prognosis compared with those with low H3K4me3 levels (P=6.8×10^-5^; **Fig. 3A**). The relationship between H3K4me3 level and patient prognosis was examined by various subset analyses. In patients with high levels of H3K4me3, stage III-IV have a worse prognosis than stage I-II patients (P=2.98×10^-4^; **Fig. 3B**). In addition, patients in stage III-IV, who had a high level of H3K4me3, had a worse prognosis than those with a low level of H3K4me3 (P=2.2×10^-3^; **Fig. 3C**). The univariate analysis in this study showed that high TNM stage, poor differentiation and high H3K4me3 levels were related to a shorter OS. In the multivariate analysis, differentiation, tumor stage, and H3K4me3 levels were considered as an independent risk factor for overall patient survival (**Table 2**).

## Discussion

H3K4 methylation is an important histone methylation 22, which has been demonstrated to be linked with several diseases, such as nerve disorders and tumors 23. Recently, H3K4 methylation has been studied more extensively in tumor progression; for example, a previous study reported its prognostic value in liver cancer. In this study, we first examined the level of H3K4me3 in adjacent normal tissues of esophageal and esophageal cancer tissues by western blotting and immunohistochemistry. Compared with adjacent tissues, the level of H3K4me3 in HEC was significantly increased, which is consistent with previous studies in liver and breast cancer 24; moreover, we found that H3K4me3 staining in HEC tissues was heterogeneous. In addition, we linked H3K4me3 level to the malignant clinicopathological features of HEC, such as the poor tumor differentiation and high tumor stage. By Ing4 interference, we indirectly identified that high levels of H3K4me3 promote the cell proliferation, colony formation, metastasis and invasion. Importantly, we showed that patient with a high level of H3K4me3 had a poor prognosis than those with a low level of H3K4me3. Thus, we concluded that the level of H3K4me3 is a valuable predictor of survival in patients with HEC.

Our results indicated that high levels of H3K4me3 promote HEC progression. Similarly, H3K4me3 was found to be related to patient survival and tumor recurrence in early-stage colon cancer in previous studies 25, and H3K4me3 was demonstrated to be a key regulator of glioma carcinogenesis 26. In addition, H3K4me3 inhibitors were found to overcome the drug resistance of pancreas ductal adenocarcinoma (PDAC) 27. Thus, our study showed that H3K4me3 level is an important and potential prognostic biomarker for screening patients with poor prognosis of HEC patients.

In summary, we have revealed a new histone modification that can be used as a biomarker for predicting prognosis for HEC patients.

## Supplementary Material

Supplementary table.

## Figures and Tables

**Figure 4:**
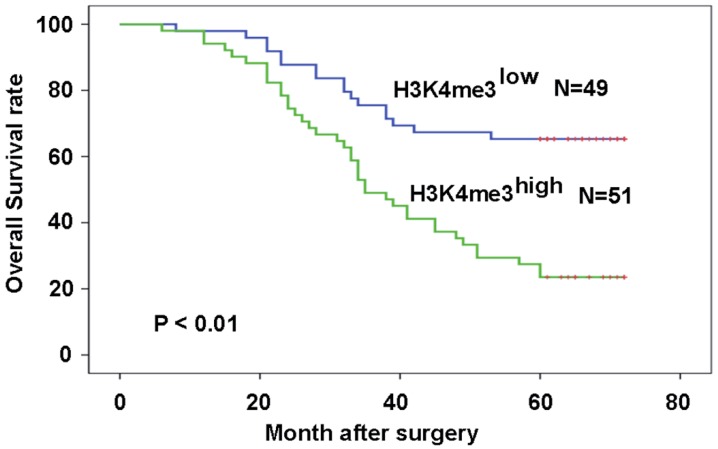
** High levels of H3K4me3 are associated with the poor prognosis of HEC patients.** Patients with high H3K4me3 levels possessed a more unfavorable prognosis compared with the patients with low H3K4me3 levels.

**Table 1:** Correlation between H3K4me3 and clinicopathological characteristics for 100 HEC patients.

Variables	Number of patients		H3K4me3 level		*P*
	100		low	high	
**Age (years)**					
≤65	74		38	36	1.00
>65	26		11	15	
**Gender**					
Male	73		33	40	0.177
Female	27		16	11	
**Localization**					
Upper	22		9	13	0.627
Middle	44		22	22	
Lower	34		18	16	
**Tumor stage**					
I-II	62		40	22	8.5×10^-5^
III-IV	38		9	29	
**Differentiation**					
Well	32		29	3	1.39×10^-5^
Moderate	43		16	27	
Poor	25		4	21	

Abbreviations: TNM, tumor-nodes-metastases. * P value < 0.05 was considered statistically significant. The Pearson Chi-square test was used.

**Table 2:** Univariate and multivariate analysis of factors associated with OS.

Variables	Univariate analysis				Multivariate analysis		
	HR	95% CI	p		HR	95% CI	p
Gender (male *vs.* female)	1.076	0.595-1.944	0.809				
Age (years) (≤65 *vs.* >65)	0.995	0.972-1.018	0.659				
Location (upper/ middle *vs.* lower)	1.033	0.716-1.492	0.862				
Tumor stage (III-IV *vs.* I-II)	2.790	1.642-4.742	1.49×10^-4^		2.067	1.153-3.705	0.015
Differentiation(well/ moderate *vs.* poor)	1.875	1.327-2.650	3.7×10^-4^		1.176	0.746-1.853	0.483
H3K4me3 level (low *vs*. high)	3.000	1.691-5.321	1.72×10^-4^		2.142	1.058-4.334	0.034

Abbreviations and note: OS, overall survival; 95% CI, 95% confidence interval; multivariate analysis, Cox proportional hazards regression model. Variables were adopted for their prognostic significance by univariate analysis with forward stepwise selection (forward, likelihood ratio). Variables were adopted for their prognostic significance by univariate analysis (p < 0.05).

## Abbreviations

H3K4me3: Histone 3 Lysine 4 tri-methylation
HEC: Human esophageal cancer
Ing4: inhibitor of growth family member 4
DAB: diaminobenzidine
BSA: bovine serum albumin
