# Supplementary material for: High level of H3K4 tri-methylation modification predicts poor prognosis in esophageal cancer
Source: J Cancer. 2020 Mar 5;11(11):3256–63. doi: 10.7150/jca.36801 (PMC7097960; doi:10.7150/jca.36801)
Supplement: Supplementary file 1 — Supplementary table. [file jcav11p3256s1.pdf]

Table S1. The ING4 shRNA Target sequence

shRNA	Sequence
ING4	GTGGTTACTCCAACCTCTA
